# 

**Published:** 1841-01

**Authors:** 


### BRITISH AND FOREIGN

### MEDICAL REVIEW

OR

### QUARTERLY JOURNAL

OF

### PRACTICAL MEDICINE AND SURGERY

LIBRARY OF [illegible]
Orleans Parish Medical Society

EDITED BY

JOHN FORBES M.D. F.R.S.

---

VOL. XI.

JANUARY—APRIL 1841

LONDON
JOHN CHURCHILL PRINCES STREET SOHO.
MDCCCXLI.

PRINTED BY C. ADLARD, BARTHOLOMEW CLOSE.

### BOOKS RECEIVED FOR REVIEW.

ENGLISH BOOKS.

1. Remarks on the Surgical Practice of Paris. Illustrated by cases. Being a Thesis to which a gold medal was assigned by the Senatus Academicus of the Edinburgh University, at the graduation of 1840. By W. O. Markham, M.D.—London, 1840. 8vo, pp. 114. 5s.

2. Derangements, primary and reflex, of the Organs of Digestion. By Robert Dick, M.D. — Edinburgh, 1840. 8vo, pp. 384. 7s. 6d.

3. A practical Treatise on the Cure of Strabismus or Squint, by operation and by milder treatment; with some new views of the anatomy and physiology of the muscles of the human eye. By P. Bennett Lucas, Surgeon. Illustrated by Plates.—London, 1840. 8vo, pp. 91. 6s.

4. A practical Treatise on the Diseases peculiar to Women, illustrated by Cases derived from hospital and private practice. By S. Ashwell, M.D., Obstetric Physician and Lecturer to Guy's Hospital. Part I. —London, 1840. 8vo, pp. 208. 7s.

5. Guy's Hospital Reports, No. XI. Oct. 1840. 8vo, Plates. 6s.

6. On the Nature and Treatment of Stomach and Urinary Diseases: being an enquiry into the Connexion of Diabetes, Calculus, and other affections of the Kidney and Bladder, with Indigestion. By Wm. Prout, M.D., F.R.S. 8vo, pp. 484. 20s.

7. The Cyclopædia of Practical Surgery. Edited by W. B. Costello, M.D. Part VII. —Cancer, Chondritis. 5s.

8. The Invalid's Guide to Madeira, with a description of Teneriffe, Lisbon, &c. By W. W. Cooper, M.R.C.S. — London, 1840. 12mo, pp. 116. 4s.

9. The Elements of Materia Medica; comprehending the Natural History, preparation, properties, composition, effects, and uses of medicines. Part II. containing the Vegetable and Animal Materia Medica. By Jon. Pereira, F.R.S.—London, 1840. 8vo, pp. 879. 24s.

10. A Treatise on the Structure, Functions, and Diseases of the Foot and Leg of the Horse. By W. C. Spooner, M.R.V.C. —London, 1840. 8vo, pp. 337. 7s. 6d.

11. A Practical Treatise on the Bilious remittent Fever; its causes and effects: with illustrative Tables and Cases on the temperature of the system in the diseases of Jamaica. By W. Arnold, M.D.—London, 1840. 8vo, pp. 320. 10s.

12. Human Physiology. By John Elliotson, M.D. Cantab. F.R.S., with which is incorporated much of the elementary part of the Institutiones Physiologicæ, of J. F. Blumenbach. M.D., F.R.S. Illustrated with numerous woodcuts. Fifth edition. —London, 1840. 8vo, pp. 1194. £2 2s.

13. Second Annual Report of the Registrar-General of Births, Deaths, and Marriages in England. — London, 1840. 8vo, pp. 247.

14. Elements of Chemistry. By the late E. Turner, M.D., F.R.S., &c. Seventh edition. Edited by Justus Liebig, M.D., and W. Gregory, M.D. — London, 1840. 8vo, pp. 988. 21s.

15. Vital Statistics of Scarborough. By John Dunn, Surgeon. — London, 1840, 4to, pp. 11.

16. A Discourse on the Phenomena of Sensation, as connected with the mental, physical, and instinctive faculties of man. By James Johnstone, M.D., Physician to the Birmingham General Hospital, &c. London, 1841. 8vo, pp. 264. 8s.

17. A Treatise on the Nervous Diseases of Women; comprising an enquiry into the nature, causes, and treatment of spinal and hysterical disorders. By Thomas Laycock, M.D. — London, 1840. 8vo, pp. 356. 10s. 6d.

18. Practical Observations on the Treatment of Stricture of the Urethra, with Cases. By Robert Wade, Surgeon to the Westminster General Dispensary, &c. —London, 1841. 8vo, pp. 149. 5s.

19. The Pathology and Diagnosis of Diseases of the Chest, comprising a rational exposition of their signs. By Charles J. B. Williams, M.D., Professor of Medicine, University College, &c. — London, 1840. 8vo, pp. 331. 10s. 6d.

20. Medical Notes and Reflections. By Henry Holland, M.D., F.R.S., &c. Second Edition.—London,1840. 8vo, pp. 638. 18s.

21. A Lecture on Toxarthus or Club Foot. By J. D. Mütter, M.D.—Philadelphia, 1839. 8vo, pp. 104.

22. The Elements of Geology, for Popular Use. By Charles A. Lee, M.D., &c. —New York, 1840. 12mo, pp. 375.

23. Thesis on the Nature and History of Plague, as observed in the North Western Provinces of India, for which a gold medal was awarded by the University of Edinburgh. By Frederick Forbes, A.M., M.D., of the Bombay Army.—Edinburgh, 1840. 8vo, pp. 102.

24. The Anatomy of the Arteries of the Human Body; with its applications to Pathology and operative Surgery. In lithographic drawings, with practical commentaries. By Richard Quain, Professor of Anatomy in University College. With delineations by Joseph Maclise, Esq, Surgeon. Parts I, II, and III. 15 Plates, super royal folio. Letter press, 8vo, pp. 112. —London, 1840. 12s. each Part.

25. Willis's Illustrations of Cutaneous Disease. Parts XX, XXI. 5s. each Part.

26. Practical Remarks on the New Operation for the cure of Strabismus or Squinting. Illustrated with lithographic Engravings. By G. W. Duffin, M.D. &c. —London, 1840. 8vo, pp. 147. 6s.

27. An Address to the Medical Practitioners of Ireland on the subject of Vaccination. Second Edition. By S. B. Labalt, M.D., &c.—Dublin, 1840. 8vo, pp. 202. 6s.

28. The Sciences accessory to Medicine essential to its successful Cultivation, the relations of these to one another, and the delights that attend the zealous study of each; a discourse delivered at the Aldersgate School of Medicine, as an introduction to the Session of 1840-41. By R. Willis, M.D., &c. London, 1840. 8vo.

29. Practical Remarks on the Discrimination and Appearances of Surgical Disease, &c. By John Howship, Surgeon to Charing Cross Hospital. London, 1840. 8vo, pp. 420. 10s. 6d.

30. Demonstrations of Anatomy; being a Guide to the Dissection of the Human Body. By G. V. Ellis, one of the Demonstrators of Anatomy in University College. London, 1840. 8vo, pp. 620. 10s.

31. An Enquiry into the Efficiency of Digitalis, in the Treatment of Idiopathic Epilepsy. By E. Sharkey, A.B., M.D.— London, 1841. 8vo. pp. 80. 4s.

32. Outlines of a Course of Lectures on Medical Jurisprudence. By T. S. Traill, M.D., Professor of Medical Jurisprudence in the University of Edinburgh. Second Edition.—Edin. 1840. 8vo, pp. 222. 5s.

33. Dr. Ramsbotham's Atlas of Plates, illustrative of Obstetric Medicine. Parts X, XI, XII. 1s. 6d. each Part.

34. Spinal Diseases: with an improved plan of Cure. Including what are commonly called Nervous Complaints. By J. H. Robertson, M.D.—Glasgow, 1841. 8vo, pp. 160.

35. Fifth Report of the Inspectors of the Prisons of Great Britain. III. Southern and Western District. By Bissett Hawkins, M.D.—London, 1840. Fol. pp. 149.

36. An Introductory Lecture on Surgery. By James Miller, one of the Surgeons of the Royal Infirmary, Edinburgh.—Edin. 1840. 8vo, pp. 23.

37. On Scientific Medicine, and its relations to claims upon Society at large: an address read before the North of England Medical Association at Carlisle, on the 15th Sept. 1840. By William Elliot, M.D.—Carlisle, 1840. 8vo, pp. 44.

38. A Treatise on the Sympathetic Relation between the Stomach and the Brain; and, throughout, between the Digestive and the Nervous Systems, in the causation and cure of Diseases. By Chas. Wightman, M.D.—London, 1840. 8vo, pp. 192. 5s. 6s.

39. Elements of Chemistry, including the most recent discoveries and applications of the science to Medicine and Pharmacy, and to the Arts. By R. Kane, M.D., M.R.I.A., &c. Part I.—Dublin, 1840. 8vo, pp. 356. 6s.

40. Descriptive Catalogue of the preparations in the Museum of the Royal College of Surgeons, Ireland. By John Honslow, M.D., M.R.I.A. Vol. II. Pathology, —Dublin, 1840. 8vo, pp. 604.

41. Practical Surgery; with 150 Engravings on wood. By Robert Liston. 3d Edition.—London, 1840. 8vo, pp. 592. 22s.

